# Groundwater Vulnerability Assessment of the Pingtung Plain in Southern Taiwan

**DOI:** 10.3390/ijerph13111167

**Published:** 2016-11-23

**Authors:** Ching-Ping Liang, Cheng-Shin Jang, Cheng-Wei Liang, Jui-Sheng Chen

**Affiliations:** 1 Department of Nursing, Fooyin University, Kaohsiung City 831, Taiwan; sc048@fy.edu.tw (C.-P.L.); cherry951753@yahoo.com.tw (C.-W.L.); 2 Department of Leisure and Recreation Management, Kainan University, Taoyuan City 338, Taiwan; csjang@mail.knu.edu.tw; 3 Graduate Institute of Applied Geology, National Central University, Taoyuan City 320, Taiwan

**Keywords:** NO_3_^−^-N, groundwater, vulnerability, DRASTIC model, discriminant analysis

## Abstract

In the Pingtung Plain of southern Taiwan, elevated levels of NO_3_^−^-N in groundwater have been reported. Therefore, efforts for assessing groundwater vulnerability are required as part of the critical steps to prevent and control groundwater pollution. This study makes a groundwater vulnerability assessment for the Pingtung Plain using an improved overlay and index-based DRASTIC model. The improvement of the DRASTIC model is achieved by reassigning the weighting coefficients of the factors in this model with the help of a discriminant analysis statistical method. The analytical results obtained from the improved DRASTIC model provide a reliable prediction for use in groundwater vulnerability assessment to nitrate pollution and can correctly identify the groundwater protection zones in the Pingtung Plain. Moreover, the results of the sensitivity analysis conducted for the seven parameters in the improved DRASTIC model demonstrate that the aquifer media (A) is the most sensitive factor when the nitrate-N concentration is below 2.5 mg/L. For the cases where the nitrate-N concentration is above 2.5 mg/L, the aquifer media (A) and net recharge (R) are the two most important factors.

## 1. Introduction

Groundwater provides a vitally important and reliable component of the water supply because of its large storage capacity and the relative cleanliness of the water quality as compared to the surface water. However, groundwater in shallow aquifers is more vulnerable to contamination by hazardous chemicals seeping down from the land surface. Farming activities and other land uses can lead to deterioration in the quality of the groundwater by introducing substantial amounts of nutrients for crops such as nitrate. Groundwater nitrate pollution is already a major concern for sustainable aquifer management in most agricultural areas worldwide. Increasing efforts are being made in many countries to protect the groundwater quality by implementing measures to prevent the pollution of groundwater by agricultural contaminant sources and by promoting good farming practices. Efforts have therefore been made in many countries to designate nitrate-vulnerable zones. High levels of nitrate concentrations in drinking groundwater can threaten human health, and this is thus widely regarded as one of the most common toxicological problems worldwide [1]. Nitrates at elevated concentrations can disintegrate within the body to form nitrites, which hamper oxygen transfer by binding hemoglobin, resulting in methemoglobinemia, which is particularly dangerous and can be harmful to infants [1]. Nitrate is considered a precursor to the development of the genotoxic N-nitroso compounds (NOC), which are known animal carcinogens [2]. Nitrate in drinking water may also enhance several types of cancer risk through the production of N-nitroso compounds in the body, which are highly carcinogenic [3].

Characterizing groundwater vulnerability can help decision makers and water management agencies to describe the areas that are more vulnerable to contamination from sources released on the ground surface, and prioritize the areas where more intensive monitoring might be needed, as well as implementing agricultural management policies, evaluating current land use practices, or adopting new measures to better prevent or control groundwater pollution. The existing approaches developed for characterizing groundwater vulnerability can be generally classified as follows: (1) process-based simulation methods; (2) statistical models; and (3) overlay and index methods [4]. In the process-based simulation methods, mathematical models are used to predict the complicated processes and mechanisms of subsurface flow and contaminant transport and the simulation results can be used to evaluate the groundwater vulnerability. However, the process-based simulation methods are reliant upon a sufficient number of hydrogeological parameters and extensive computational efforts, thus restricting their applications for groundwater vulnerability assessment [5]. Generally speaking, one-dimensional models are most commonly used within the process-based simulation method. The application of a statistical method to evaluate the relationship between the actual occurrence of pollutants in the groundwater and the spatial variables generally requires extensive water quality monitoring, accurate data, and cautious determination of the spatial variables. Overlay and index methods are able to consider the parameters that control the migration of pollutants from the land surface into the shallow aquifers. In the overlay and index method, the controlling physical parameters are indexed and assigned appropriate weights from which to calculate the composite index for assessing the groundwater vulnerability. The main advantage is the ease of use, low data requirements, and clear explanation of groundwater vulnerability. However, the subjectivity involved in specifying numerical values to the grade of the descriptive parameters and relative weights for different parameters seems to be its main drawback. A variety of comprehensive overlay and index methods have been proposed for determination of groundwater vulnerability. Among these, the DRASTIC model [6,7] developed by a committee of the U.S. Environmental Protection Agency (EPA) is the most well-established method worldwide despite some significant disadvantages that have been identified in its applications.

The DRASTIC model is not used to absolutely measure the groundwater vulnerability but rather provides a reference standard for decision making. For example, Babiker et al. [8] evaluated groundwater vulnerability in the Kakamigahara aquifer, which is situated in the southern part of the Gifu Prefecture of central Japan, using the DRASTIC model with the help of the geography information system (GIS) platform. They found that the net recharge parameters had the largest impact on aquifer vulnerability, followed by soil media, topography, vadose zone media, and hydraulic conductivity. It was found that the net recharge and hydraulic conductivity were the two most effective parameters for the groundwater vulnerability assessment. Similarly, Baalousha [9] used a DRASTIC model to assess the groundwater vulnerability of the Gaza Strip, Palestine. They found the obtained groundwater vulnerability map to be positively correlated to the known pollution values. Assaf and Saadeh [10] compared the groundwater nitrate contamination against the vulnerability index from the DRASTIC model. They indicated that the vulnerability index from the DRASTIC model was frequently in good agreement with the observed groundwater nitrate contamination levels. However, the DRASTIC model has received several criticisms, especially because the selected factors and weights do not always meet the hydrological and geological characteristics of the studied area, frequently resulting in the low reliability of the groundwater vulnerability assessment [9,11].

A number of researchers have attempted to improve the assessment reliance of the groundwater vulnerability. For example, Antonakos and Lambrakis [12] improved the DRASTIC model by incorporating the logistic regression approach for assessing groundwater vulnerability to nitrates in Northeast Korinthia, Greece. Saidi et al. [13] reassigned the weights of the seven parameters in the DRASTIC model to modify the prediction reliability of the groundwater vulnerability assessment. Huan et al. [14] developed an improved DRASTIC model to evaluate the groundwater vulnerability to nitrate pollution in Jilin City of northeastern China. They rebuilt the parameter index system, adjusting the grade scale of each parameter index, specifying new index weights, and comparing the grading methods for groundwater vulnerability to nitrate pollution to reduce the subjectivity of the DRASTIC model. More recently, Jang et al. [15] developed an improved model for assessing the groundwater vulnerability for the Choushui River alluvial fan in western Taiwan using a discriminant analysis (DA) approach.

**Figure 1:**
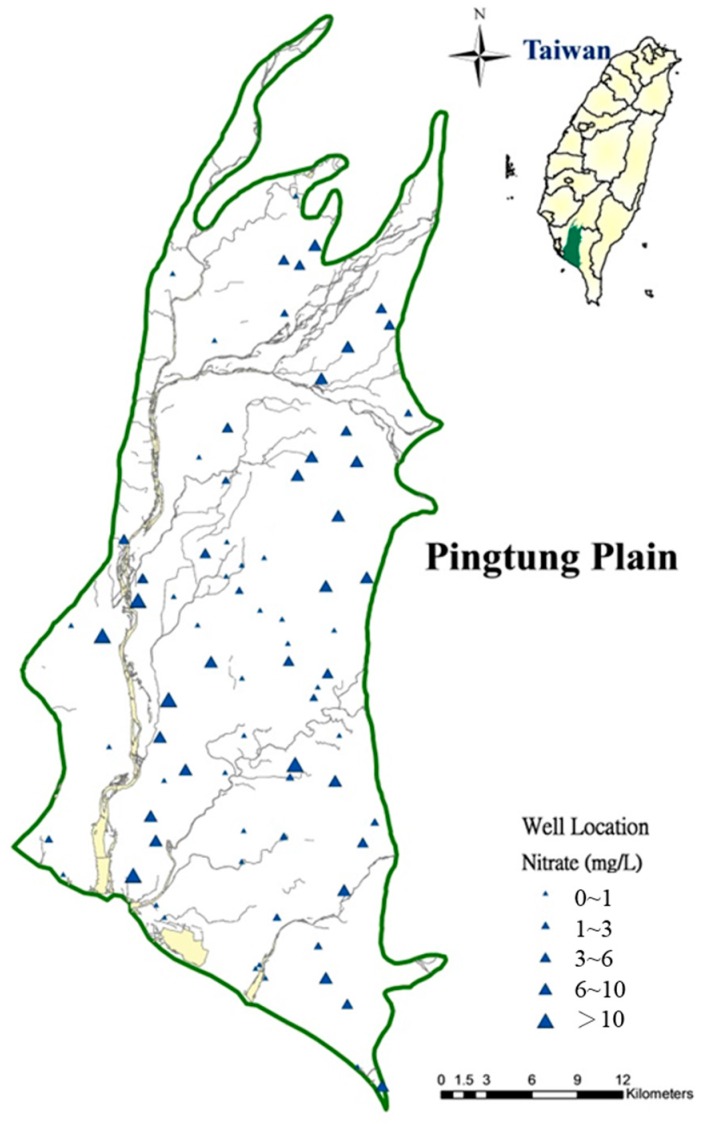
Spatial distribution of nitrate concentrations. The triangles size represents nitrate concentrations of <1, 1–3, 3–6, 6–10, and >10.0 mg/L, respectively.

The Pingtung Plain is an important area for agricultural production in Taiwan, with approximately 45.7% of the land used for agriculture. It is unusual that only approximately 45.8% of inhabitants of the Pingtung Plain use tap water treated at a water plant (the average percentage of population served of the tap water in Taiwan is 92.93%). People in the Pingtung Plain use a substantial amount of groundwater to meet drinking and household needs. In recent years, nitrate contamination has been identified as a major source of groundwater contamination in Taiwan. Elevated levels of the NO_3_^−^-N in groundwater have been reported based on long-term groundwater quality monitoring [16]. Figure 1 displays the spatial distribution of nitrate concentrations on the Pingtung Plain.

To protect the groundwater as an important water resource in the Pingtung Plain, it is necessary to evaluate which locations are more vulnerable to pollution. Therefore, this study attempts to comprehensively assess groundwater vulnerability in the Pingtung Plain using an overlay and index-based DRASTIC model. To overcome the restrictions of the traditional DRASTIC model, it is improved by the incorporation of DA analysis to promote the reliability of predictions about groundwater vulnerability to nitrate pollution.

## 2. Materials and Methods

### 2.1. Study Area

**Figure 2:**
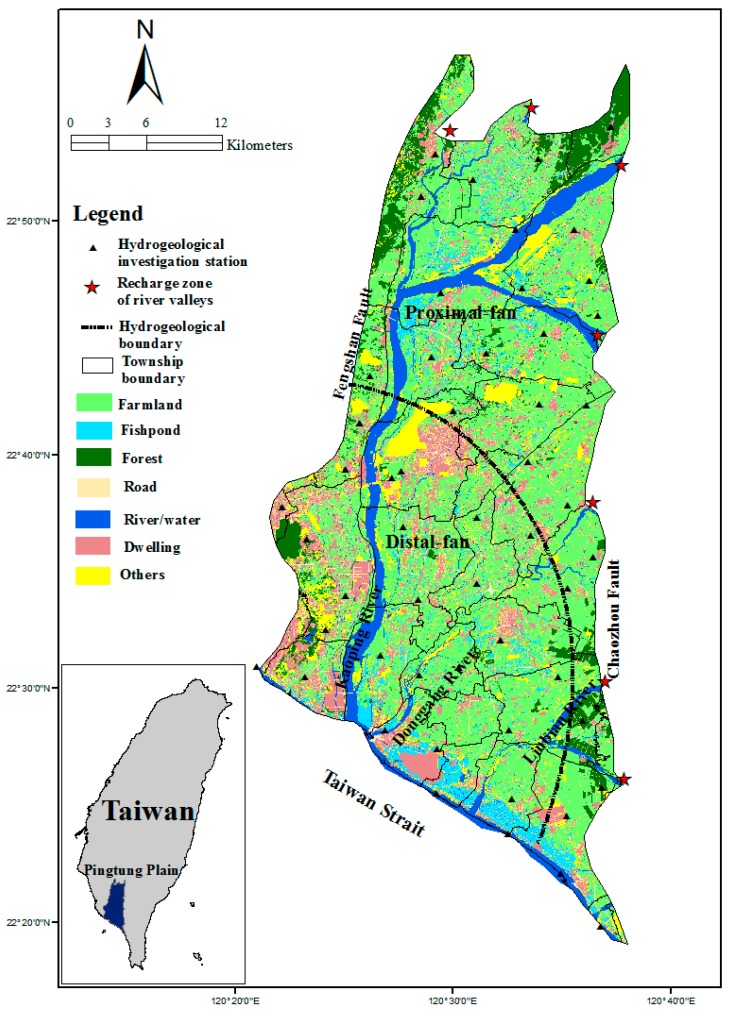
Study area and land use.

Our study area is the Pingtung Plain, located in southern Taiwan. The plain includes most of Pingtung County and part of Kaohsiung City and consists of 25 townships (Figure 2). It is bound by foothills and river valleys to the North, the Fengshan Fault to the West, the Taiwan Strait to the South, and the Chaozhou Fault to the East and has a total area of 1210 km^2^. The average rainfall is 2493 mm per year, with most of the precipitation during the period from May to September and much less precipitation during the winter months of October to December and spring months of January to April. The Kaoping River is the main river crossing the Pingtung Plain with the largest drainage area in Taiwan. Two shorter rivers, the Dongkang and Linbian Rivers, also flow through this plain.

**Figure 3:**
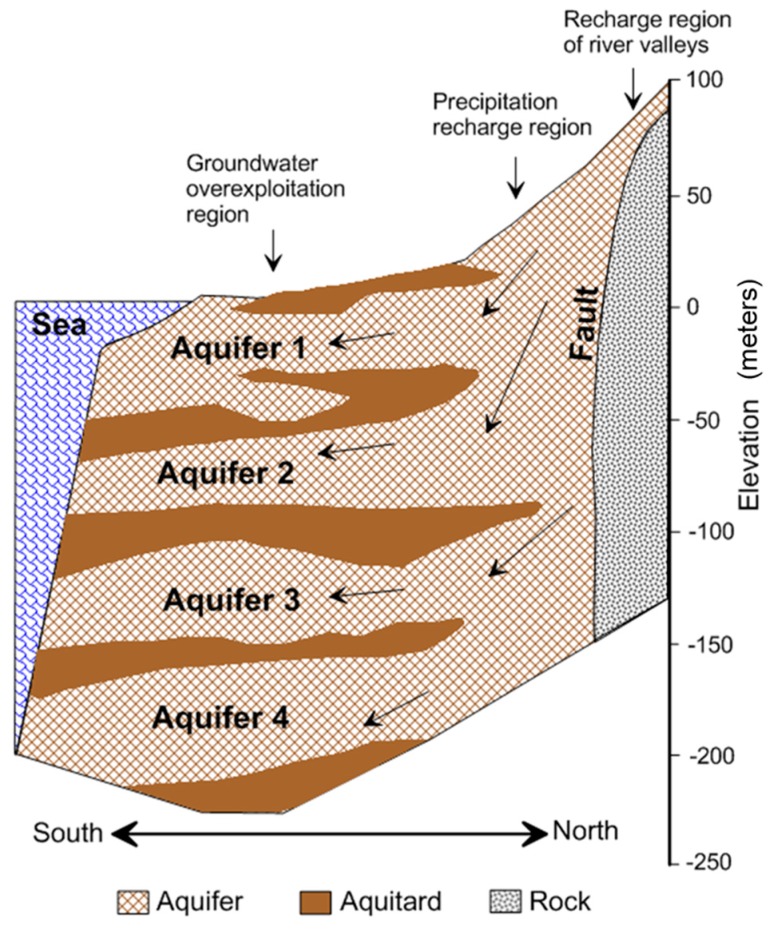
Hydrogeological profile.

The unconsolidated sediments of the Late Pleistocene and the Holocene contain abundant groundwater that has resulted in a reliance on irrigation through the legal and illegal extraction of groundwater. Most of the sediments consist of coastal and estuarine sand and mud, with abundant shallow marine and lagoon shells and foraminifers. This plain is partitioned primarily into a proximal fan and a distal fan. Analyses of the subsurface geology and hydrogeology were conducted from 1995 to 1998. Subsurface hydrogeological analysis to a depth of approximately 250 m divided the plain deposits in the distal fan into eight overlapping sequences, including four marine sequences and four non-marine sequences [17]. Non-marine sequences with coarse sediments are identified as aquifers, whereas marine sequences with fine sediments are considered as aquitards [18] (Figure 3). Aquitards are located mainly in the distal fan but not present in the proximal fan. Natural annual rainfall infiltration replenishes the groundwater and is the primary source of fresh water on the plain. The proximal fan and the river valleys at the eastern and northern boundaries are the major regions for aquifer recharging.

The main uses of land in the study area are for the cultivation of crops and for aquaculture. Land use for agriculture accounts for approximately 45.7% of the study area, whereas 5.1% of the land is used for fish ponds. During dry months and years, substantial amounts of groundwater are extracted to meet the demand for farmlands, fish ponds, and households, leading to an increase in groundwater salinity, a reduction of the pollution-diluting capability of the surface water, and a rise in the occurrence of severe land subsidence and seawater intrusion in the Pingtung Plain [19].

### 2.2. DRASTIC Model

A DRASTIC model that is constructed with the aid of a GIS environment is used to evaluate the groundwater vulnerability of the Pingtung Plain. The DRASTIC model is developed based on the concept of the geological setting, which is defined as a composite description of all the major geologic and hydrological factors that govern and affect the movement of the pollutant into the groundwater. The acronym DRASTIC is derived from seven parameters: **D**epth to water, net **R**echarge, **A**quifer media, **S**oil media, **T**opography, **I**mpact of vadose zone, and hydraulic **C**onductivity. The DRASTIC model uses a numerical index that is obtained by combining the ratings and weights assigned to the seven model parameters. The significant media types or classes of each parameter reflect the ranges, which are rated from 1 to 10 according to their relative impact on groundwater vulnerability. The seven parameters are then assigned weights from 1 to 5, reflecting their relative importance. The DRASTIC index is calculated from a linear combination of the product of the ratings and weights of seven parameters as follows.

(1) $$\mathrm{DRASTIC}\;\mathrm{Index}\;(\mathrm{DI})=D_{r}D_{w}+R_{r}R_{w}+A_{r}A_{w}+S_{r}S_{w}+T_{r}T_{w}+I_{r}I_{w}+C_{r}C_{w},$$

where D, R, A, S, T, I, and C are the seven parameters and the subscripts r and w indicate the corresponding ratings and weights, respectively. The greater the DRASTIC index, the higher the groundwater vulnerability.

### 2.3. Discriminant Analysis

Discriminant analysis is a multivariate method and has comprehensive applications in the study of environmental problems. In this study, it is used to determine the weights of the seven hydrogeological factors in the modified model by comparing the actual nitrate-N concentration and the computed results of the DRASTIC model. DA is frequently used to determine a linear combination of parameters that characterizes or separates two or more classes of objects or events [20]. DA has two functions: classification and prediction. It is used to partition events (cases) into the values of a categorical dependent variable. In DA, a discriminant function is constructed for each group ($G_{i}$) as follows:

(2) $$f(G_{i})=k_{i}+\sum_{j=1}^{n}w_{ij}p_{ij},$$

where i is the number of groups $G_{i}$, $k_{i}$ is a constant inherent to each group, n is the number of parameters that is used to divide a set of data into a given group ($G_{i}$), $p_{ij}$ is the *j^th^* parameters for a given group ($G_{i}$), and $w_{ij}$ is a weighting coefficient assigned by DA to a given parameters $p_{ij}$. DA is based on a prior knowledge of samples separated into various categories in advance. Classifications using DA objectively characterize the relationship between the major parameters through the linear combination of the hit ratio, which is defined as the ratio of correctly classified numbers to the total number. A greater hit ratio represents better classification. In this study, the nitrates concentrations at the monitored wells were classified into two groups, polluted and unpolluted, in advance based on the observed nitrate presence. DA is used to build a discriminant model in which the seven factors of the DRASTIC models are considered. The classification performance is graded based on the area under the curve (AUC) of the receiver operating characteristic (ROC). The correct classification is used to measure the prediction performance. The correct classification ratio is defined as a ratio of the true positive and true negative to the total number.

### 2.4. Data Preparation

Several types of data are required to construct the ratings and weights of the seven parameters in the DRASTIC model. The depth to water table (*D*) is obtained by subtracting the groundwater level from the elevation of the well. The average monthly groundwater levels between 2006 and 2011 are taken from long-term observations carried out by the Taiwan Water Resources Agency (WRA). Following Babiker et al. [8], the net recharge (*R*) is computed from the rainfall data using the following equation:

(3) Net Recharge=(Rainfall−Evapotranspiration)×recharge ratio.

Ten rainfall stations were built by the Taiwan Central Weather Bureau (CWB) in the study area. Generally speaking, the recharge in the Pingtung Plain is primarily due to direct infiltration by precipitation. The rainfall distribution is generated by spatial interpolation of the annual precipitation as obtained from the rainfall stations. Considering that there are only three evaporation stations in the study area, the spatial variability of evaporation is neglected and the spatially-invariant average of evaporation data observed at the two stations used for the entire study area. Moreover, the recharge ratios were determined based on different land use types with reference to Chow et al. [21] (see Table 1). The ratings of the aquifer media (A) and soil media (S) are both based on borehole data showing soil texture, published by the Central Geological Survey (CGS), Taiwan. Fifty-one sets of borehole data are used in this study. The slope of the topography (T) is estimated using the elevation as derived from a digital terrain model (DTM). Moreover, borehole data from the CGS of Taiwan is used to compute the impact of the vadose zone (I). The hydraulic conductivity (C) is obtained based on hydraulic tests performed in aquifer 1 and published by the CGS. The data on the actual nitrate concentration are obtained from the WRA. The average nitrate concentration was 2 mg/L, and the standard deviation was 0.79 mg/L. Approximately 21.7% of the samples exceeded the acceptable threshold values of the drinking water standard in Taiwan. The study area was divided into “polluted” and “unpolluted” areas based on the actual nitrate concentration thresholds. Three distinct nitrate-N thresholds of 2.5, 5.5, and 7.5 mg/L are used to map the individual groundwater protection zones.

## 3. Results and Discussion

### 3.1. Ratings of Parameters and Vulnerability Index Obtained Using the Conventional DRASTIC Model

The ratings of the parameters *D*, *R*, *T*, and *C* in the DRASTIC model are based on the standards set by the U.S. EPA [6] and Aller et al. [7]. While parameters *A*, *S*, and *I* are closely related to the hydrological conditions, the hydrological settings that would be the case for the U.S. should differ from those for the alluvial fan in Taiwan. Thus, we use the standard soil texture categories developed by the Taiwan CGS [22] to determine the ratings of these three factors. The eight categories of soil textures include the following: coarse gravel (cG), gravel (G), fine gravel (fG), coarse sand (cS), medium sand (mS), fine sand (fS), mud (M), and clay (C). The ratings for the seven parameters used in the DRASTIC model are summarized in Table 2.

**Figure 4:**
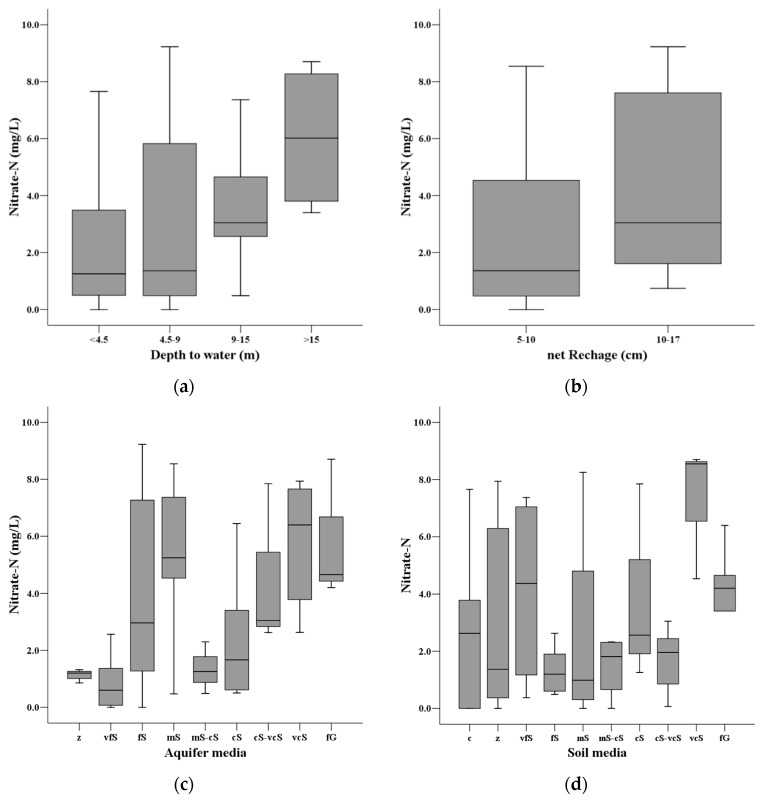

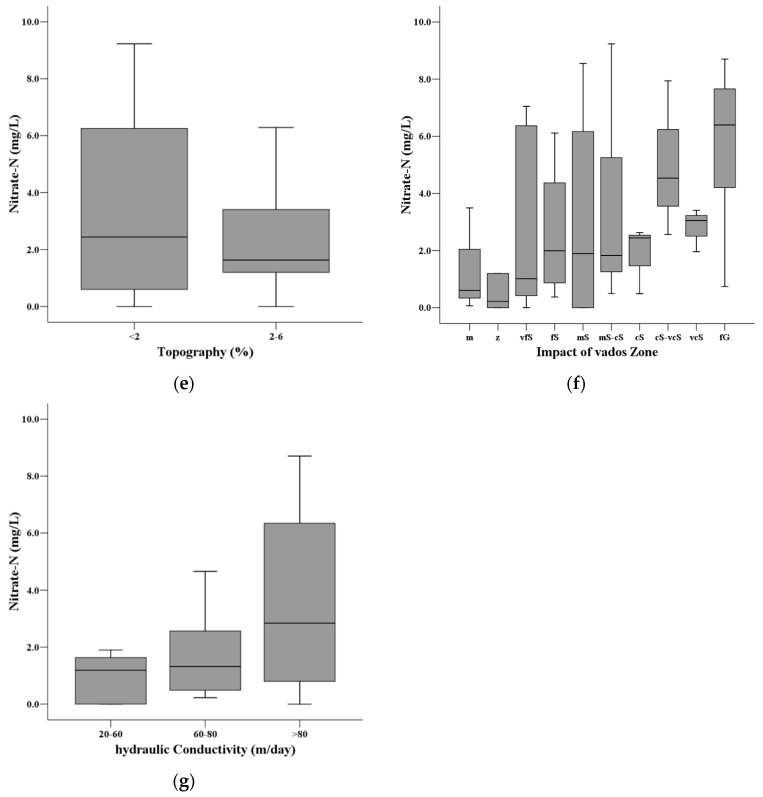
Box plots showing the distribution of groundwater nitrate concentration for the statistically different classes for the seven parameters in the DRASTIC model. (**a**) Depth to water; (**b**) net recharge; (**c**) aquifer media; (**d**) soil media; (**e**) topography; (**f**) impact of vadose zone; (**g**) hydraulic conductivity. after the DRASTIC model.

The ratings of each factor plotted against the actual NO_3_^−^-N concentration are depicted with box-and-whiskers plots in Figure 4. It can be seen that the distribution of the actual nitrate concentration is wider for factor *R* for high ratings, whereas *S* and *T* tend to have wider distributions for low ratings. Factor *C* shows a wide distribution for high and low ratings and a narrow distribution for medium ratings.

**Figure 5:**
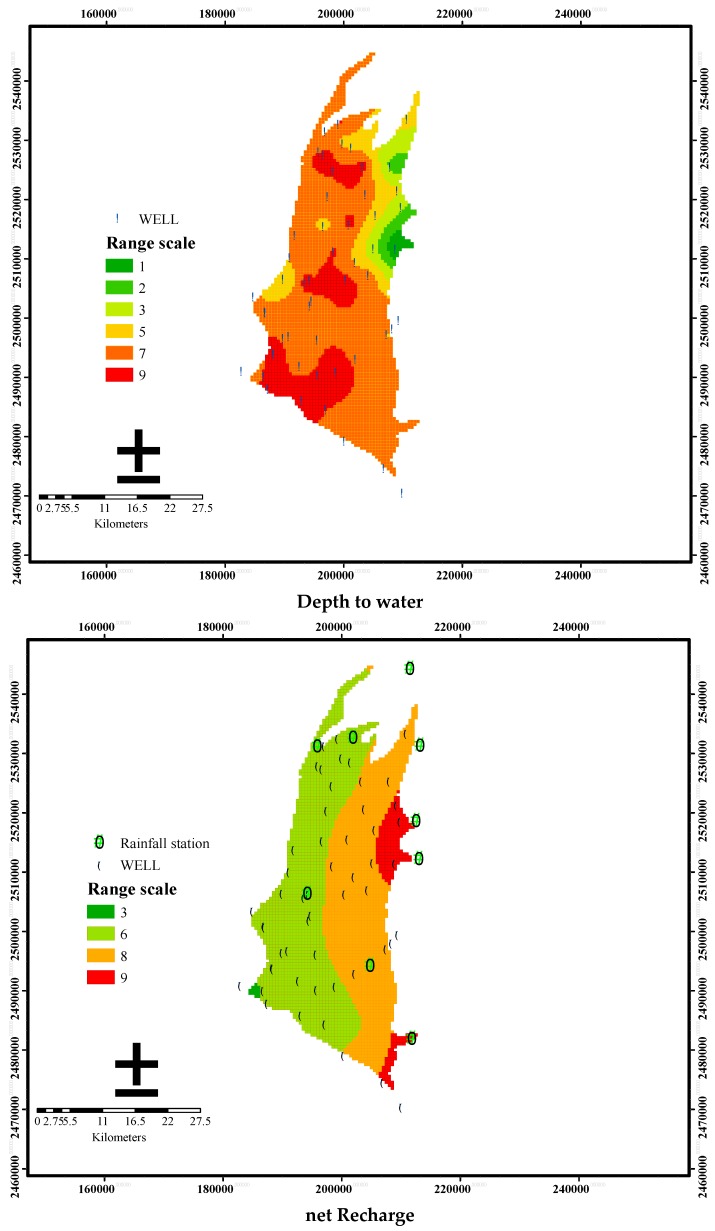

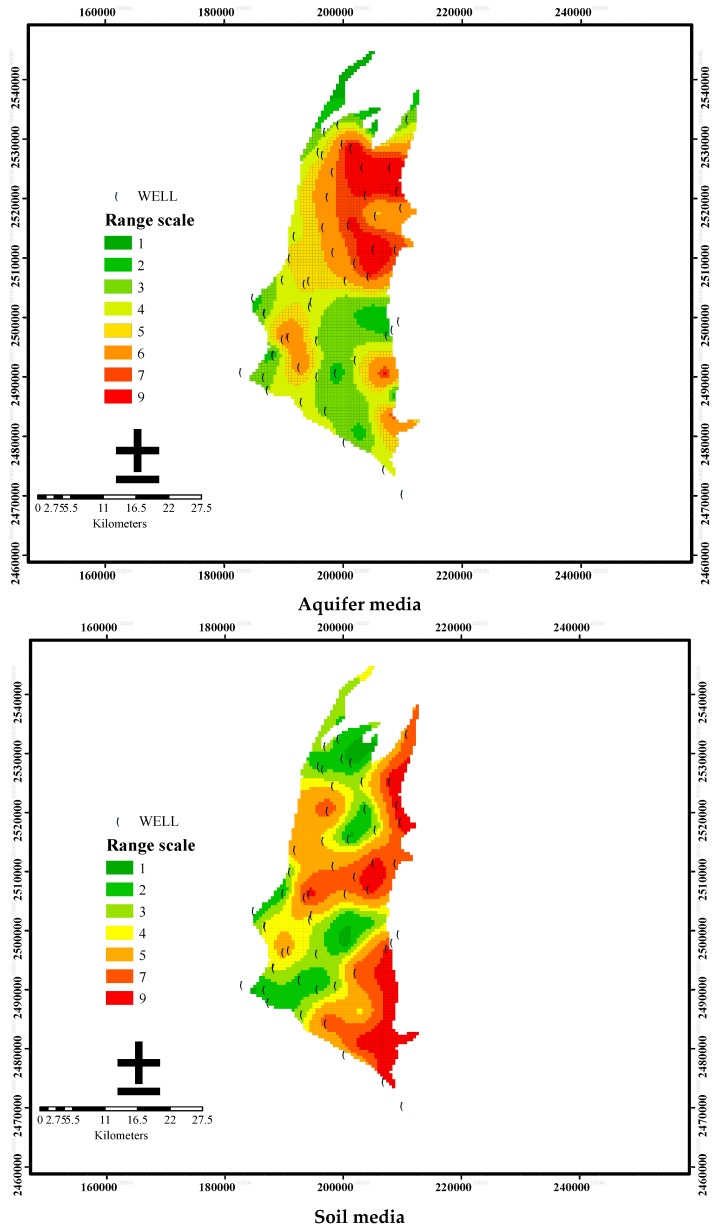

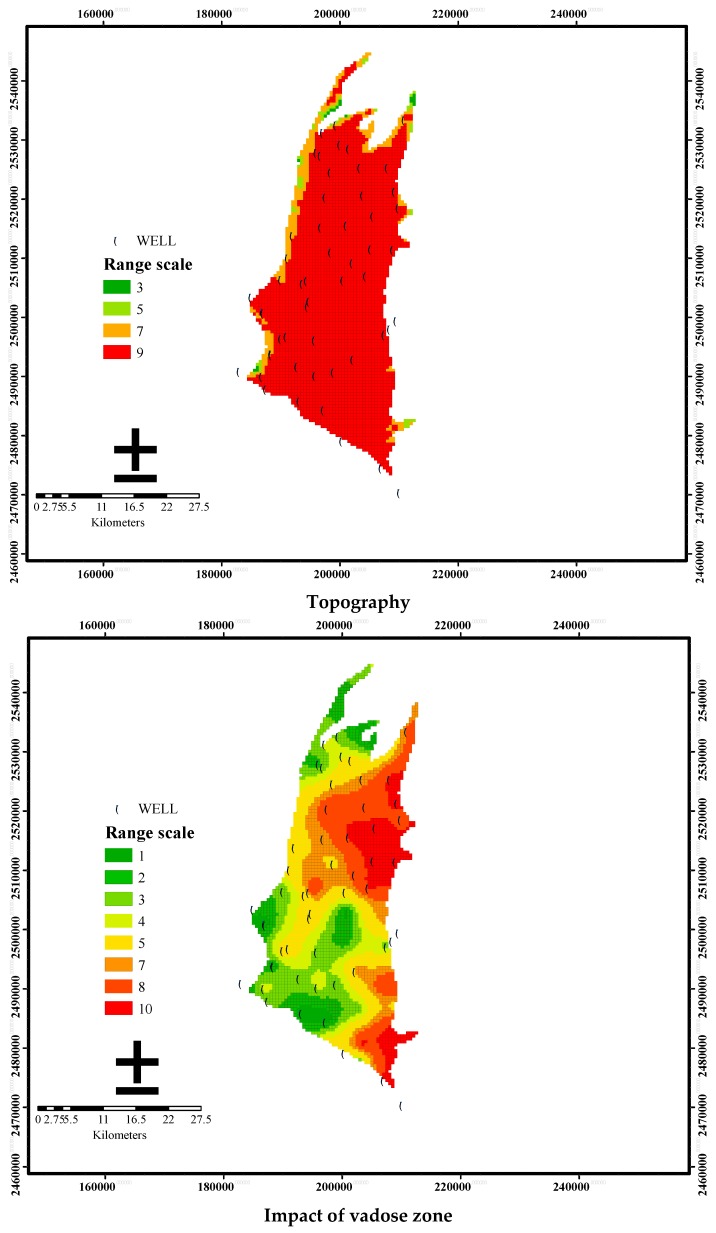

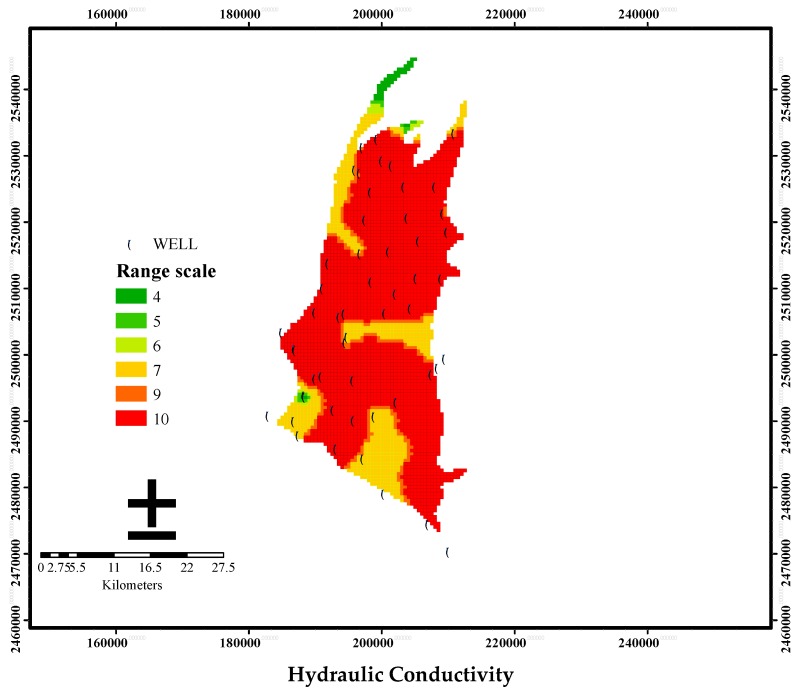
Spatial distribution of the ratings for the seven factors of the DRASTIC model in the Pingtung Plain. **D**: **D**epth to water; **R**: net **R**echarge; **A**: **A**quifer media; **S**: **S**oil media; **T**: **T**opography; **I**: **I**mpact of vadose zone; **C**: hydraulic **C**onductivity.

The spatial distribution of the ratings for each factor in the DRASTIC model is depicted in Figure 5. Parameters *R*, *A*, *S*, *I*, and *C* show “high” ratings in the eastern part of the study area. For parameter *D*, high ratings are found in the distal fan and mid-fan areas. Parameter *T* shows high rating in the eastern foothills and low ratings in the plain regions. The vulnerability index is then calculated using Equation (1) with the specified ratings and weights of the seven parameters as shown in Table 1.

**Figure 6:**
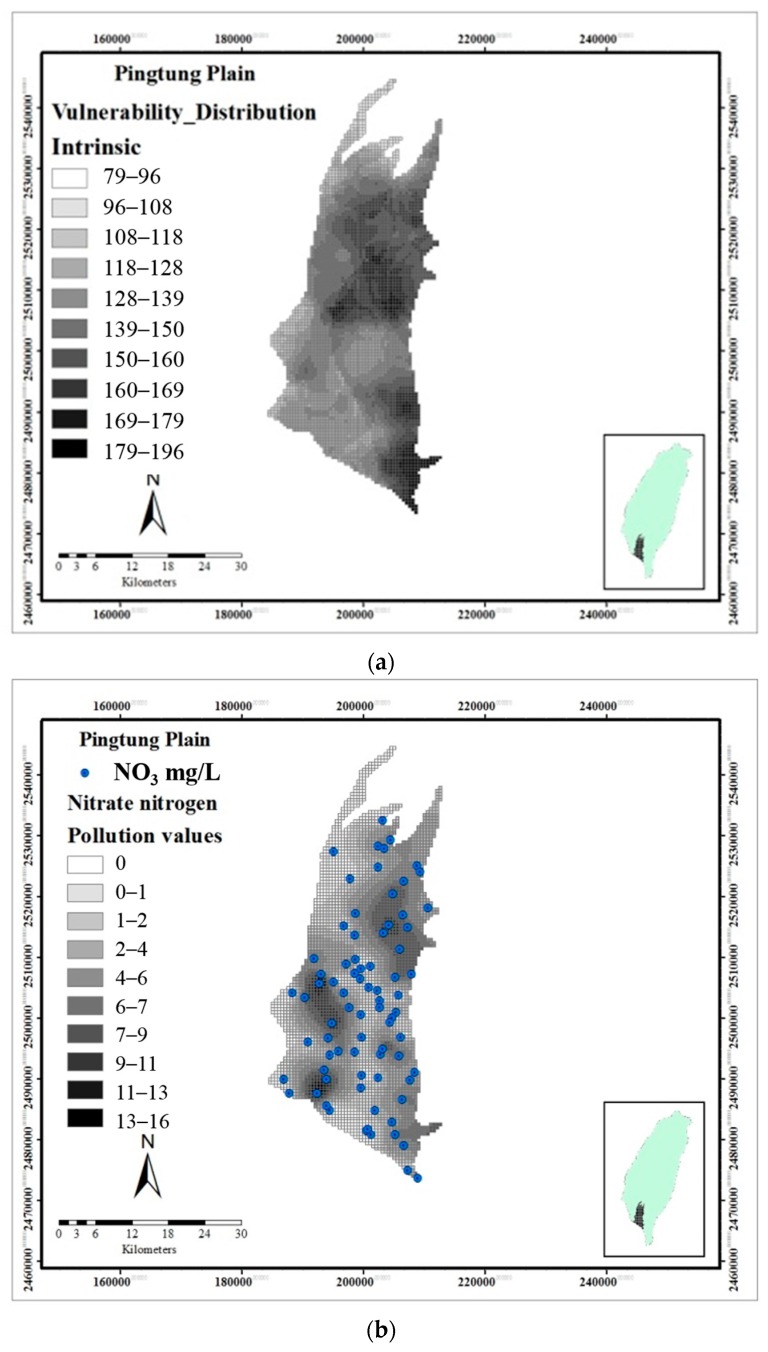
Comparison of the spatial distribution of (**a**) DRASTIC vulnerability index and (**b**) nitrate concentrations.

The spatial distributions of the ratings are obtained based on spatial interpolation using the Kriging method [23] and the SURFUR software. The study area was discretized with a spacing of 1000 × 1000 m. The spatial distribution of the DRASTIC index ranges between 79 and 196, as depicted in Figure 6. The central and southeastern areas of the Pingtung Plain are attributed “high vulnerability”, while the eastern part shows a “moderate vulnerability” class. The southwestern part of the study area displays “low vulnerability” based on the final DRASTIC score. The actual NO_3_^−^-N concentration is often selected as an indicator for the assessment of groundwater vulnerability and pollution risk [10,24]. The application of the DRASTIC model depends on the correlation between the actual NO_3_^−^-N concentration and the computed vulnerability index. The actual NO_3_^−^-N concentration concentrations are obtained from 78 shallow groundwater samples that were collected from 2006 to 2011. It can be noted that the actual NO_3_^−^-N nitrate concentration and the vulnerability index do not correlate with each other very well.

### 3.2. Improved DRASTIC Model Using Discriminant Analysis

As noted, the actual nitrate concentration and the results of the groundwater vulnerability assessment do not correlate with each other very well. From the results, we can conclude that the conventional DRASTIC model cannot be used to correctly represent the actual groundwater vulnerability. Several studies have also demonstrated that there is a poor correlation between the computed vulnerability index obtained using the conventional DRASTIC model with the actual NO_3_^−^-N concentrations [9,10,12]. The correlation between the actual NO_3_^−^-N concentrations and the computed vulnerability index obtained using the conventional DRASTIC model is 0.17. In order to enhance the predictive reliability of groundwater vulnerability, the DA approach is adopted herein and used to reassign the weights of the seven parameters in the DRASTIC model based on the actual nitrate concentration. We consider three nitrate concentration thresholds of 2.5, 5.5, and 7.5 mg/L to characterize the three different levels of NO3^−^-N pollution. Unpolluted and polluted groups are categorized based on the three distinct nitrate concentration thresholds. 

The discriminant functions for three distinct concentration thresholds of 2.5, 5.5 and 7.5 mg/L using DA are as follows:

(3a) Function 1=−3.00−0.17D+0.02R+0.32A−0.11S+0.13T+0.10I+0.14C

(3b) Function 2=−7.45+0.01D+0.55R+0.18A−0.32S+0.42T+0.12I+0.12C

(3c) Function 3=−4.50+0.08D+0.52R+0.32A−0.19S+0.36T+0.12I−0.31C.

**Figure 7:**
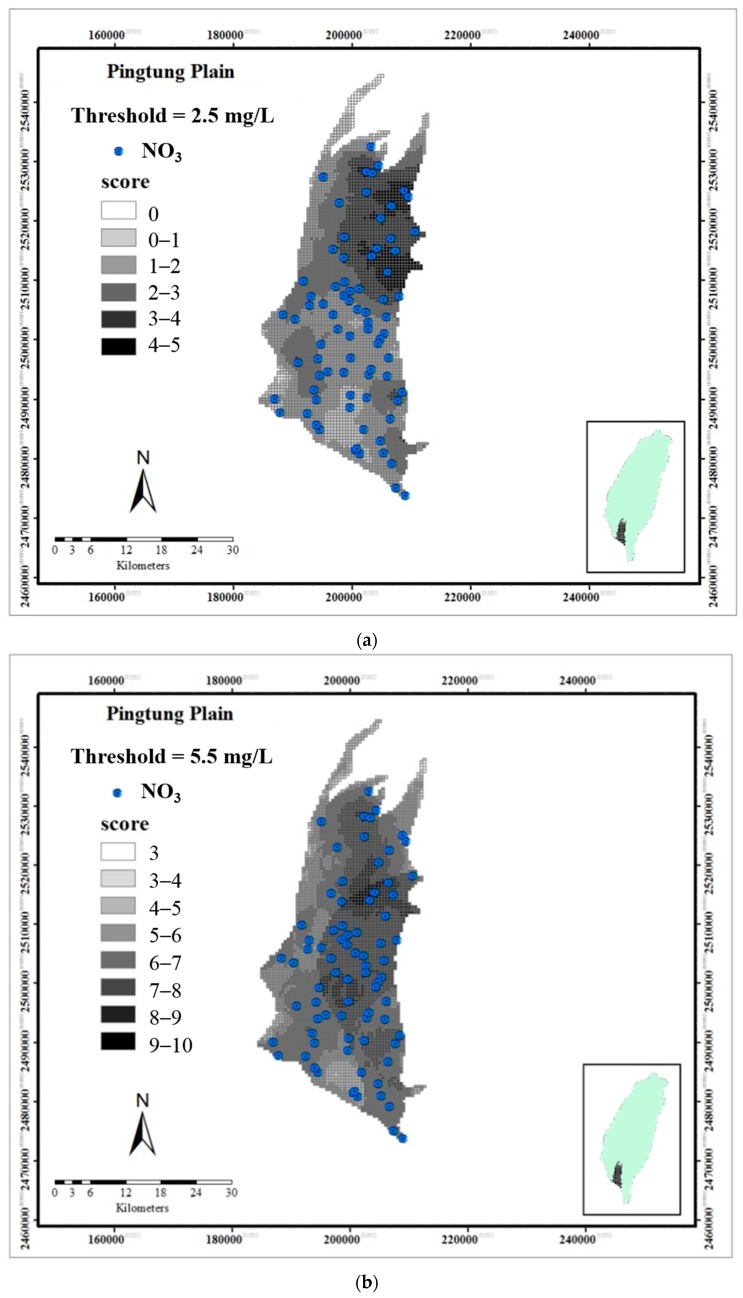

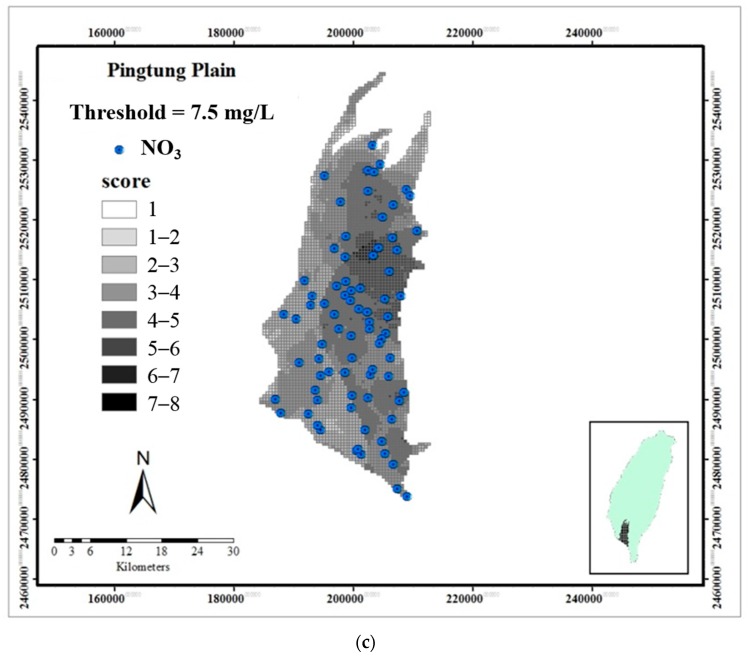
Spatial distribution of the vulnerability index obtained from the modified DRASTIC model for three distinct thresholds: (**a**) 2.5; (**b**) 5.5; and (**c**) 7.5 mg/L.

The results from the modified DRASTIC model using DA illustrate a linearly increase in the DRASTIC index with the factors of *R*, *A*, *T*, and *I* and show a linear decrease with *S*. Table 3 compares the correct classification ratios and AUCs obtained from the traditional and modified DRASTIC model. The total correct classification ratios for three concentration thresholds are enhanced from 64%, 60%, and 64% for the conventional DRASTIC model to 74%, 75%, and 79% for the improved DRASTIC model. The AUCs for the three concentration thresholds are elevated to 0.84, 0.80, and 0.84 for the improved DRASTIC model as compared with 0.71, 0.63, and 0.76 of AUCs for the conventional DRASTIC model. This clearly demonstrates the superiority of the improved DRASTIC model over the conventional one. Figure 7 depicts the vulnerability index obtained from the improved DRASTIC model for three distinct concentration thresholds, 2.5, 5.5, and 7.5 mg/L. The groundwater protection zones can be delineated based on these figures to implement an effective management scheme for the agriculture fertilizer utilization. Moreover, to decrease the health risk of nitrate exposure to humans, groundwater utilization should be limited in highly vulnerable regions.

### 3.3. Sensitivity of the Factors Used in DRASTIC

After determining the corresponding discriminant functions for the three nitrate concentration thresholds, we further performed sensitivity analysis to identify the relative importance of these factors. First, we calculated the arithmetic mean for each parameter for both the polluted and unpolluted groups. Then the calculated average values for each factor were substituted for the three discriminant functions in Equations (3a)–(3c). The absolute differences for each factor for both groups were adopted to assess how sensitive the parameters in the obtained discriminant functions are. Table 4 summarizes the absolute value of the difference between the unpolluted and polluted groups for each parameter subject to three NO_3_^−^-N thresholds of 2.5, 5.5, and 7.5 mg/L. For an NO_3_^−^-N threshold of 2.5 mg/L, the aquifer media (*A*) has the most important influence on the groundwater vulnerability, followed by the impact of vadose zone (*I*). The net recharge (*R*) and aquifer media (*A*) are the two most sensitive factors for the NO_3_^−^-N threshold of 5.5 mg/L and 7.5 mg/L. Jang et al. [15] performed the groundwater vulnerability assessment for Choushui River alluvial fan in central Taiwan. They concluded the depth to groundwater (*D*) and aquifer media (*A*) are the two most sensitive factors for the NO_3_^−^-N level of 3.0 mg/L and the impact of vadose zone play the most important role for the NO_3_^−^-N level of 5.0 mg/L. The relative importance of the model parameter is different in the two areas of Taiwan. This study used the modified DRASTIC model to obtain a more reliable prediction than the traditional DRASTIC model. Sensitivity analysis of the factors in the modified DRASTIC model could indicate the significant contributing factors for different levels of nitrate pollution. This approach can be used to explore major causes of nitrate pollution based on groundwater vulnerability in other aquifers.

## 4. Conclusions

An improved DRASTIC model is developed for characterizing groundwater vulnerability for the Pingtung Plain in southern Taiwan. The discriminant analysis statistical method is adopted to revise the weights of the seven parameters used in the improved DRASTIC model by the comparison between the vulnerability index and the actual NO_3_^−^-N concentrations. The results demonstrate that the improved DRASTIC model has more reliable assessment accuracy than the conventional DRASTIC model. Sensitivity analysis of the seven parameters in the improved DRASTIC model illustrates that aquifer media are the critical parameters for the three different concentration thresholds. The net recharge is also the most important parameters for nitrate concentration thresholds of 2.5 mg/L and 5.5 mg/L. The spatial distribution of groundwater vulnerability to nitrate concentration, as delineated by the modified DRASTIC model, can provide a reliable vulnerability map to help government decision makers adopt appropriate measure to prevent and control groundwater pollution in the Pingtung Plain.

## Figures and Tables

**Table 1:** The recharge ratios for different land use types. (Modified from Chow et al. [21]). The recahge ratio is calculated based on the runoff data for different land use types provided by Chow et al. [21].

Land Use Types	Recharge Ratio
Developed area	
Road/building roof	0.10
Park/lawn	0.55
Undeveloped	
Cultivated land	0.60
Pasture/range	0.65
Forest/woodlands	0.70

**Table 2:** Weights, ranges, and ratings for the seven parameters in the DRASTIC model.

	D_(m)_^a^	R_(cm)_^a^	A ^b^	S ^b^	T ^a^	I ^b^	C_(Cmd/m_^2^_)_^a^
**Weights**	5	4	3	2	1	5	3
**Ratings**							
**10**	0–1.5	-	vcG, cG, mG, fG	vcG, cG, mG, fG	0–2	vcG, cG, mG, fG	81>
**9**	1.6–4.5	>26	vcS	vcS	3–6	vcS	-
**8**	-	18–25	-	-	-	-	41–80
**7**	4.6–9.0	-	cS	cS	-	cS	-
**6**	-	11–17	-	-	-	-	29–40
**5**	9.1–15	-	mS	mS	7–12	mS	-
**4**	-	-	fS	fS	-	fS	13–28
**3**	16–22.5	6~10	vfS	vfS	13–18	vfS	-
**2**	22.6–30	-	Z	Z	-	Z	4–12
**1**	31+	0–5	M, C	M, C	19+	M, C	<4

^a^ the ratings proposed by Aller et al. [7]; ^b^ cG: coarse gravel; G: gravel; fG: fine gravel; cS: coarse sand; mS: medium sand; fS: fine sand; M: mud; C: clay.

**Table 3:** Summary of results obtained by the discriminant analysis.

Thresholds (mg/L)	Total Correct Classification Ratios (%)	Correct Classification Ratios of Polluted Groups (%)	Correct Classification Ratios of Unpolluted Groups (%)	AUC
Intrinsic DRASTIC model				
2.5	64	62	66	0.71
5.5	60	62	60	0.63
7.5	64	86	61	0.76
Modified DRASTIC model				
2.5	74	63	83	0.84
5.5	75	69	78	0.80
7.5	79	86	78	0.84

AUC: area under the curve.

**Table 4:** Absolute difference for each factor between the polluted and unpolluted groups for the three distinct nitrate-N thresholds of 2.5, 5.5, and 7.5 mg/L.

**Group Types (2.5 mg/L)**	**−0.17D**	**0.02R**	**0.32A**	**−0.11S**	**0.13T**	**0.10I**	**0.14C**
**Polluted**	−0.98	0.08	2.51	−0.59	1.29	0.78	1.39
**Unpolluted**	−1.31	0.08	1.09	−0.51	1.26	0.43	1.26
**Difference**	0.331	0.002	1.426	0.082	0.030	0.348	0.130
**Group types (5.5 mg/L)**	**0.01D**	**0.55R**	**0.18A**	**−0.32S**	**0.42T**	**0.12I**	**0.12C**
**Polluted**	0.07	2.66	1.22	−1.49	4.18	0.88	1.15
**Unpolluted**	0.07	1.84	0.76	−1.60	4.06	0.56	1.10
**Difference**	0.006	0.820	0.464	0.107	0.121	0.318	0.043
**Group types (7.5 mg/L)**	**0.08D**	**0.52R**	**0.32A**	**−0.19S**	**0.36T**	**0.12I**	**−0.31C**
**Polluted**	0.50	2.54	2.32	−0.97	3.58	0.95	−2.95
**Unpolluted**	0.58	1.77	1.32	−0.91	3.48	0.55	−2.87
**Difference**	0.082	0.764	0.997	0.065	0.094	0.404	0.080

**D**: Depth to water; **R**: net Recharge; **A**: Aquifer media; **S**: Soil media; **T**: Topography; **I**: Impact of vadose zone; **C**: hydraulic Conductivity.
